# Unidirectional alteration of methylation and hydroxymethylation at the promoters and differential gene expression in oral squamous cell carcinoma

**DOI:** 10.3389/fgene.2023.1269084

**Published:** 2023-10-12

**Authors:** Weizhi Zhao, Lin Zhu, Qian Gong, Suzhen Ma, Haofeng Xiong, Tong Su, Zhengqing Wan, Danling Wang

**Affiliations:** ^1^ Institute for Future Sciences, University of South China, Changsha, Hunan, China; ^2^ The Affiliated Changsha Central Hospital, Hengyang Medical School, University of South China, Changsha, Hunan, China; ^3^ Xiangya Hospital, Central South University, Changsha, Hunan, China; ^4^ Department of Medical Genetics, Hunan Provincial Maternal and Child Health Care Hospital, Changsha, Hunan, China; ^5^ MOE Key Lab of Rare Pediatric Diseases, School of Life Sciences, University of South China, Changsha, Hunan, China

**Keywords:** oral squamous cell carcinoma, multi-omics analysis, methylation, hydroxymethylation, whole genome bisulfite sequencing, whole genome oxidative bisulfite sequencing

## Abstract

**Background:** Oral squamous cell carcinoma (OSCC) is one of the most common types of cancer worldwide. Although overall losses of 5-methylcytosine (5mC) and 5-hydroxymethylcytosine (5hmC) have been previously observed, a genome-wide, single-base-resolution, and simultaneous mapping of 5mC and 5hmC in OSCC is still unaccomplished. Similarly, the mechanism of how 5mC and 5hmC collectively lead to abnormal gene expression in OSCC is largely unexplored. Using parallel whole-genome bisulfite sequencing (WGBS) and whole-genome oxidative bisulfite sequencing (oxWGBS), we characterized 5mC- and 5hmC-profiles at single-nucleotide resolution in paired primary OSCC samples and their normal adjacent tissues (NATs). We also analyzed the effect of 5mC- and 5hmC-modifications on differential gene expression in OSCC using multi-omics analysis.

**Results:** An overall reduction of both 5mC and 5hmC in various genomic regions have been observed in OSCC samples. At promoter regions, a total of 6,921 differentially methylated regions and 1,024 differentially hydroxymethylated regions were identified in OSCC. Interestingly, compared to bidirectional modification with 5mC and 5hmC, unidirectional modification with 5mC and 5hmC at the promoters is associated with bigger change in the gene expression. Additionally, genes bearing unidirectional modification with 5mC and 5hmC at the promoters are enriched in signaling pathways like cell proliferation, cell differentiation, and receptor tyrosine kinase pathway that are essential for the tumorigenesis. Finally, the grouped expression signature of top 20 genes bearing promoter-unidirectional-modification with 5mC and 5hmC tends to correlate with the clinical outcome of certain subtypes of head and neck squamous cell carcinoma.

**Conclusion:** Using parallel WGBS and oxWGBS analyses, we observed an overall reduction of 5mC- and 5hmC-modifications at various genomic regions in OSCC. Unidirectional modification with 5mC and 5hmC at the promoters is associated with enhanced changes in gene expression in OSCC tissues. Furthermore, such differentially expressed genes bearing unidirectional modifications with 5mC and 5hmC at the promoters might have clinical relevance to the outcome of OSCC.

## 1 Introduction

DNA methylation is a main type of epigenetic modification. It involves the attachment of a methyl moiety to the DNA molecule and occurs mainly on cytosine (Smith and Meissner, 2013). Using S-adenosylmethionine as a methyl donor, DNA methyltransferases catalyze the methylation of the 5′ position on a cytosine ring to produce 5-methylcytosine (5mC) (Lyko, 2018). Cytosine methylation predominantly happens at the CpG (5′-C-phosphate-G-3′) dinucleotide sequence—in human somatic cells, more than 98% of DNA methylation occurs on CpG sites (Jin B. et al., 2011), and about 60%–80% of all CpG sites are normally methylated (Smith and Meissner, 2013). Functionally, 5mC is widely accepted as a negative regulator of gene transcription (Luo et al., 2018). The cumulative existence of 5mC eases the conformational change of DNA and facilitates the compaction of genomic DNA into a more rigid structure, retaining the DNA in a transcriptionally inactive status (Choy et al., 2010). When presenting at promoter regions, 5mC impedes the recruitment of transcriptional machinery and suppresses the initiation of transcription in a cell type-specific manner.

Although 5mC is overall stable, a small amount of 5mC can undergo removal of methyl moiety through an active, stepwise procedure: first, ten-eleven translocation (TET) proteins, also known as α-ketoglutarate-dependent dioxygenases, oxidate 5mC to 5-hydroxymethylcytosine (5hmC), 5-formylcytosine (5 fC), and 5-carboxycytosine (5caC), sequentially; then, thymine DNA glycosylase (TDG) rapidly converts 5fC and 5caC to unmethylated cytosine through a “base excision repair” mechanism to finish the demethylation process (He et al., 2011; Kohli and Zhang, 2013; Wu and Zhang, 2014). Among these demethylation intermediates, 5fC and 5caC are transient and spare, but 5hmC is relatively long-lived and abundant.

Unlike 5mC, 5hmC seems to have more perplexed effects on gene transcription. The distribution and enrichment of 5hmC have been observed at various locations in human genomic DNA with discordant associations with the changes in gene expression (Jin SG. et al., 2011; Pastor et al., 2011; Song et al., 2011; Yu et al., 2012). Positive correlation has been suggested between the gene-body 5hmC and the transcription of the marked gene since the enrichments of 5hmC in gene bodies are often associated with upregulated gene expression (Ficz et al., 2011; Wu and Zhang, 2017). However, when 5hmC is enriched at promoter regions, the marked genes are either not upregulated, or downregulated, suggesting the transcriptional regulation by promoter region’s 5hmC is likely different from that by gene body’s (Stroud et al., 2011; Wu et al., 2011; Greco et al., 2016). In addition, the involvements of 5hmC in gene expression have been noted at distal intergenic cis-regulatory elements like active enhancer, p300 binding site, and CTCF (CCCTC-binding factor) binding site (Stroud et al., 2011; Yu et al., 2012). The level of 5hmC at tissue-specific enhancers has been reported to be positively correlated with gene expression (Cui et al., 2020), but the associations between 5hmC at other cis-regulatory elements and gene expression are not fully characterized yet. Therefore, more research is needed to understand how exactly 5hmC is involved in the regulation of gene expression.

Despite the incomplete depiction of geographic distribution and functional profile of 5hmC, the involvements of 5hmC in important biological processes, such as stem cell differentiation (Ito et al., 2010; Koh et al., 2011), epigenetic reprogramming (Song et al., 2013), oxygen sensing and regulation (Mariani et al., 2014), and neuronal differentiation (Hahn et al., 2013; Lister et al., 2013), are readily evident. Pathologically, a global reduction of 5hmC has been noted in various types of human cancer, such as colorectal and prostate cancer (Kudo et al., 2012; Takayama et al., 2015), myeloid leukemia (Wernig-Zorc et al., 2019), melanoma (Lian et al., 2012), and others (Yang et al., 2013), suggesting the contribution of dysregulated 5hmC in the progress of human cancers.

Oral squamous cell carcinoma (OSCC) is the most common malignancy affecting the oral cavity and constitutes the majority of head and neck squamous cell carcinoma (HNSC) (Cramer et al., 2019). Using immunohistochemical analyses, a few research groups have reported that 5hmC is remarkably decreased in OSCC (Wang et al., 2017; Cuevas-Nunez et al., 2018); TET, the enzyme producing 5hmC, is also found decreased in OSCC, both indicating the involvement of 5hmC in the OSCC pathogenesis (Jäwert et al., 2013). Until now, a precise, genome-wide 5hmC profiling in OSCC at single-nucleotide resolution is still unavailable; consequently, a quantitative determination of 5hmC’s abundance at different genomic regions is not yet established.

In this study, we characterized the pattern of 5mC- and 5hmC-modifications in the primary OSCC samples and their paired normal adjacent tissues (NATs) using parallel whole-genome bisulfite sequencing (WGBS) and whole-genome oxidative bisulfite sequencing (oxWGBS). We found that the compound, unidirectional modification with 5mC and 5hmC at promoter regions correlates with a bigger change in the gene expression in OSCC tissues, and the suchlike differentially expressed genes may have clinical importance relevant to the disease outcome. Together, our data suggest that unidirectional modification with 5mC and 5hmC at the promoters may mark the genes playing important roles in the pathogenesis of OSCC.

## 2 Results

### 2.1 Distribution of 5mC and 5hmC across the whole genome

In this study, we first performed WGBS and oxWGBS with paired primary OSCC tissues and their NATs. Approximately 600 million clean reads were obtained from each sample. The average read depth was 28.70-fold with greater than 89.90% mapping rate in WGBS libraries and 28.96-fold with greater than 91.10% mapping rate in oxWGBS libraries (Table 1).

**TABLE 1:** Sequencing profile of WGBS and oxWGBS libraries.

	Sample	Clean reads	Clean bases	Q30 rate ^a^ (%)	Sequencing depth	Unique mapped ^b^	Unique mapping ratio (%)
WGBS libraries	OC1	569970506	82633519152	88.92	27.54	527568692	92.6
	OC2	570213290	82439467664	88.80	27.48	516872272	90.6
	OC3	568644076	82301936403	88.80	27.43	510985282	89.9
	OC4	624800250	90422075379	88.23	30.14	565639838	90.5
	NAT1	566512406	82024868069	88.77	27.34	525373400	92.7
	NAT2	566517370	81987069977	88.34	27.33	513593544	90.7
	NAT3	700909108	101433944635	88.23	33.81	645255440	92.1
	NAT4	590750532	85488542093	89.11	28.50	540144292	91.4
oxWGBS libraries	OC1	628468496	91712046044	92.44	30.57	590627810	94.0
	OC2	608550138	88404352123	92.37	29.47	556896964	91.5
	OC3	635429008	92577244650	92.79	30.86	578951570	91.1
	OC4	589652916	86050330292	91.87	28.68	542268292	92.0
	NAT1	580049946	84511764019	92.47	28.17	534226000	92.1
	NAT2	563790376	82157867968	92.50	27.39	517949550	91.9
	NAT3	581018552	84666207383	92.50	28.22	540049120	92.9
	NAT4	585005208	85048250789	92.52	28.35	540299068	92.4

^a^ Q30 rate: The percentage of base with a Phred value (Q score) > 30.

^b^ Unique mapped: The number of clean reads that are uniquely mapped onto the hg38 reference genome.

With spike-in, control DNA-duplexes carrying methylation and hydroxymethylation modifications, we confirmed the high bisulfite-conversion rates and high oxidative-bisulfite-conversion rates of our libraries (Table 2, the average bisulfite-conversion rate was 99.33% in WGBS libraries; the average bisulfite- and oxidative-bisulfite-conversion rates were 99.41% and 96.52%, respectively, in oxWGBS libraries). With a cutoff criterion of at least 10-fold coverage, 22.19–29.96 million CpG sites from each library were identified for further evaluation of their 5mC- and 5hmC-modifications (Table 3).

**TABLE 2:** Bisulfite- and oxidative-bisulfite-conversion rates in WGBS and oxWGBS libraries.

	Type	Sample								Average (%)
		OC1 (%)	OC2 (%)	OC3 (%)	OC4 (%)	NAT1 (%)	NAT2 (%)	NAT3 (%)	NAT4 (%)	
WGBS libraries	C–U	99.35	99.35	99.37	99.28	99.31	99.29	99.38	99.32	99.33
	5 fC–U	60.22	58.48	59.95	58.48	59.50	58.79	60.75	59.55	59.47
	5mC–U	3.95	3.74	3.89	3.95	3.79	3.76	3.76	3.81	3.83
	5hmC–U	4.76	4.90	4.80	5.00	4.62	4.72	4.99	5.32	4.89
oxWGBS libraries	C–U	99.37	99.42	99.39	99.45	99.41	99.37	99.46	99.39	99.41
	5 fC–U	92.22	93.12	91.81	91.25	92.55	92.39	92.41	92.42	92.27
	5mC–U	3.10	3.28	3.12	3.56	3.19	3.05	3.13	3.26	3.21
	5hmC–U	96.52	96.39	96.57	96.56	96.78	96.43	96.56	96.31	96.52

**TABLE 3:** Number of different cytosine context and mean methylation-rate in WGBS and oxWGBS libraries.

	Sample	10× CG number	CG mean rate	10× CHG number	CHG mean rate	10× CHH number	CHH mean rate
WGBS libraries	OC1	26434224	0.716	114319511	0.0056	315733149	0.0051
	OC2	27824844	0.674	109100931	0.0057	284482930	0.0052
	OC3	27887494	0.670	111051270	0.0057	292867637	0.0052
	OC4	22185753	0.685	103429258	0.0056	311839150	0.0051
	NAT1	25891353	0.465	104602353	0.0052	281982481	0.0047
	NAT2	24830851	0.569	96151681	0.0052	247836986	0.0047
	NAT3	26455030	0.450	103830781	0.0053	273450793	0.0048
	NAT4	26022189	0.520	104970705	0.0050	283730571	0.0045
oxWGBS libraries	OC1	26192832	0.060	108970910	0.0054	285857040	0.0049
	OC2	26874985	0.065	105485705	0.0053	269681543	0.0048
	OC3	26420088	0.068	103540681	0.0050	264212262	0.0046
	OC4	26264457	0.065	113311312	0.0053	311355358	0.0048
	NAT1	25472454	0.058	102166804	0.0046	267703710	0.0042
	NAT2	24657028	0.059	95693892	0.0047	242582807	0.0042
	NAT3	29964647	0.059	119553175	0.0047	318878805	0.0043
	NAT4	26537866	0.062	105684482	0.0050	277256173	0.0046

**FIGURE 1:**
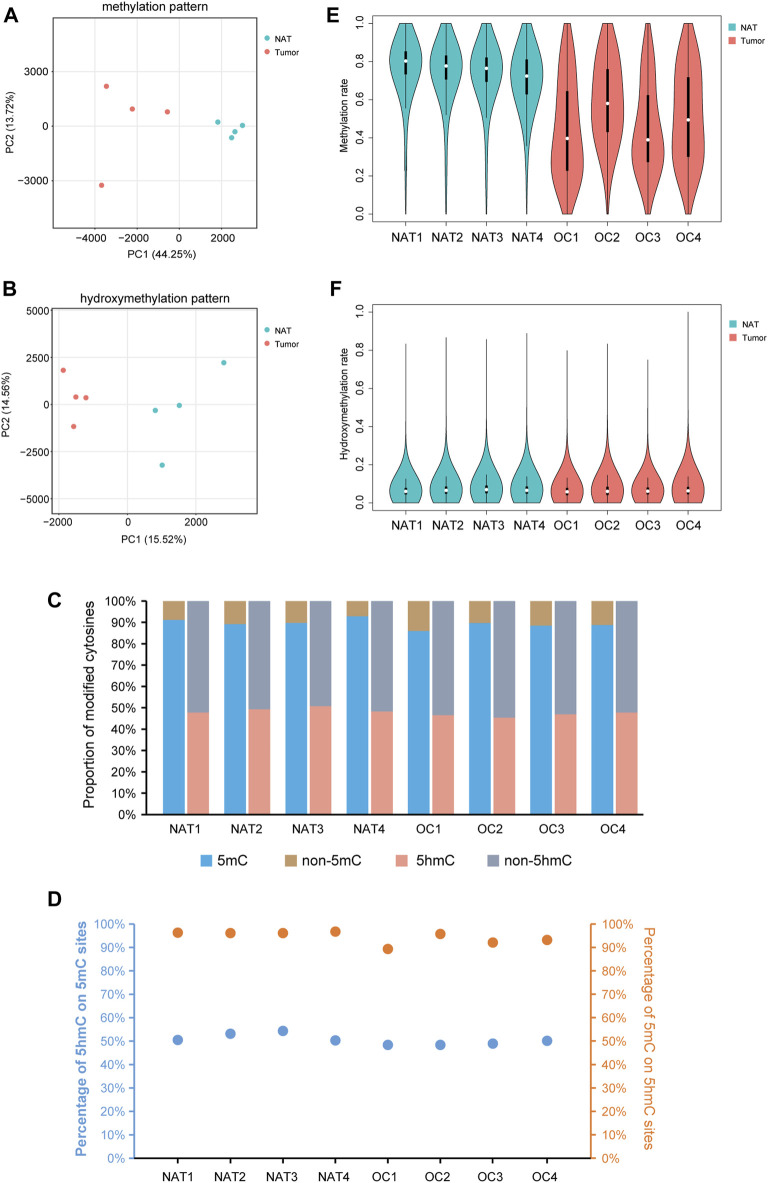
Profile of DNA methylation and hydroxymethylation in OSCC and NAT samples. **(A)**. PCA plot shows the different characteristics of DNA methylation patterns of OSCC tissues and NATs. Each dot indicates a sample. **(B)**. PCA plot shows the different characteristics of DNA hydroxymethylation patterns of OSCC tissues and NATs. Each dot indicates a sample. **(C)**. Staking bar plot shows the proportion of methylated or hydroxymethylated cytosines in different samples. **(D)**. The percentage of 5hmC in 5mC modified sites and the percentage of 5mC in 5hmC modified sites. **(E)**. Violin plot shows the overall methylation level of OSCC and NAT samples. **(F)**. Violin plot shows the overall hydroxymethylation level of OSCC and NAT samples. OSCC: oral squamous cell carcinoma. NAT: normal adjacent tissue. 5mC: 5-methylcytosine. 5hmC: 5-hydroxymethylcytosine.

Principal component analysis (PCA), together with the hierarchical clustering analysis, revealed the overall separations of 5mC- and 5hmC-data matrices between OSCCs and NATs, suggesting the underlying differences between the two conditions (Figures 1A, B, Supplementary Figure S1). Consistent with previous studies of human cells (Smith and Meissner, 2013; Azizgolshani et al., 2021), we observed 89.47% of all CpG sites bearing a certain degree of 5mC and 47.84% of all CpG sites having a certain extent of 5hmC in our samples (Figure 1C). On average, 5hmC co-presented in 50.52% of 5mC sites, and 5mC co-presented in 94.47% of 5hmC sites (Figure 1D).

Splitting the genome into 10-kb bins and plotting the level of CpG methylation of each bin, we noticed an apparent difference between the overall distributions of 5mC in OSCC and NAT groups: In NAT group, the 10-kb sized genomic regions are mainly highly-methylated, but in OSCC group, they show a much wider range with different methylation levels (Figure 1E). As shown in Figure 1F, the overall distributions of 5hmC are much alike between OSCC and NAT groups. Although not statistically significant, ratios of CpG sites containing 5mC and those containing 5hmC both appear decreased in OSCC tissues relative to NATs (Figure 1C, 88.21% of CpGs in OSCC vs. 90.74% of CpGs in NAT are methylated; 46.65% of CpGs in OSCC vs. 49.04% of CpGs in NAT are hydroxymethylated), suggesting the OSCC samples might still possess some changes in the distributions of 5mCs and 5hmCs across the genome.

### 2.2 Distribution of 5mC and 5hmC across genomic regions

**FIGURE 2:**
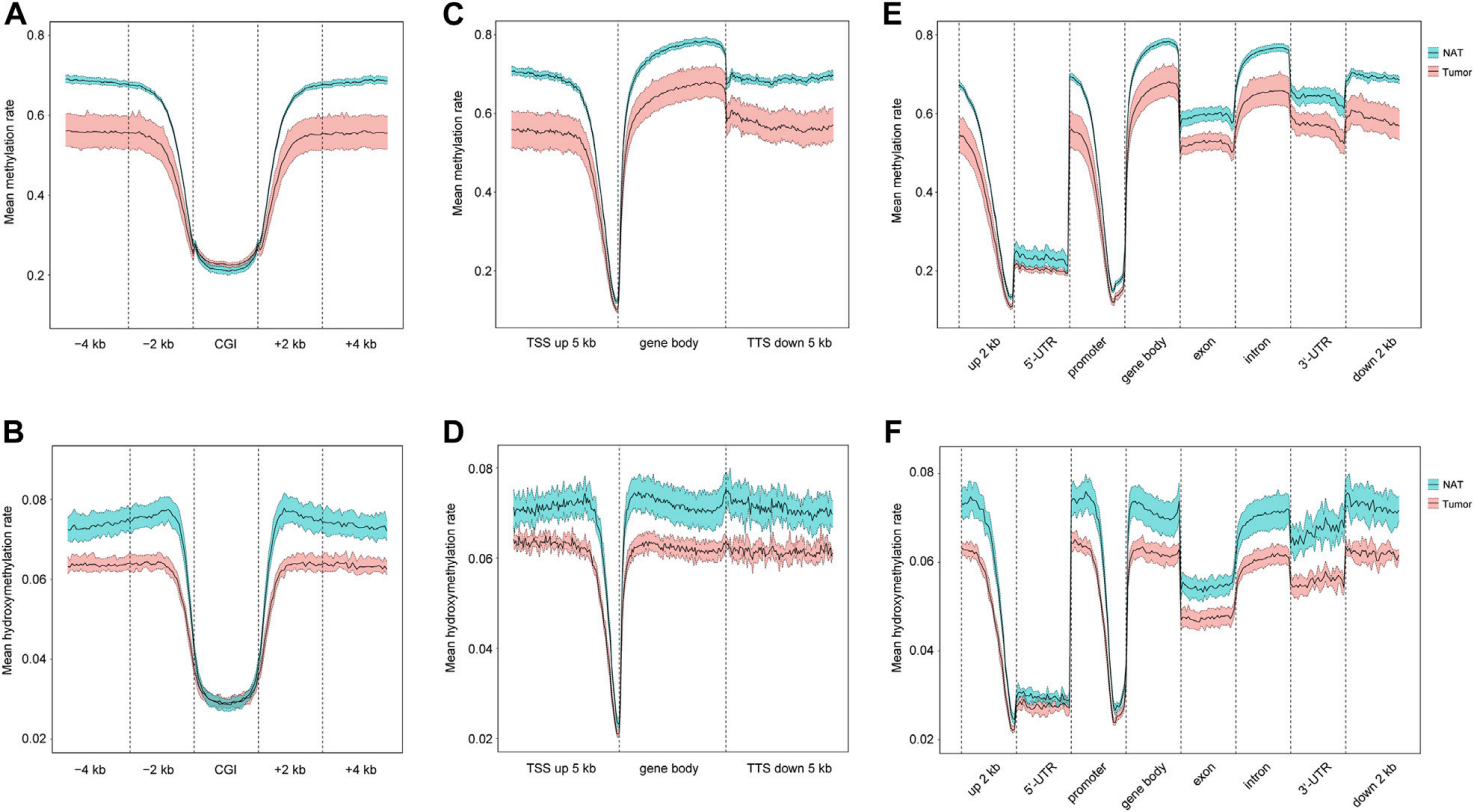
Distribution of 5mC and 5hmC in gene regulatory elements. **(A)**. Mean methylation rate at CGI, CGI ± 2 kb region, and CGI ± 4 kb region. **(B)**. Mean hydroxymethylation rate at CGI, CGI ± 2 kb region, and CGI ± 4 kb region. **(C)**. Mean methylation rate at gene body, 5 kb upstream TSSs, and 5 kb downstream TTSs. **(D)**. Mean hydroxymethylation rate at gene body, 5 kb upstream TSSs, and 5 kb downstream TTSs. **(E)**. Mean methylation rate of 5mC at 2 kb upstream 5′-UTR, 5′-UTR, promoter, gene body, exon, intron, 3′-UTR, 2 kb downstream 3′-UTR. **(F)**. Mean hydroxymethylation rate at 2 kb upstream 5′-UTR, 5′-UTR, promoter, gene body, exon, intron, 3′-UTR, 2 kb downstream 3′-UTR. Blue- and red-colored areas with the mean line represents 95% confidence intervals of NAT and tumor samples, respectively. CGIs: CpG islands. TSSs: transcription start sites. TTSs: transcription termination sites. 5′-UTR: 5′-untranslated region. 3′-UTR: 3′-untranslated region.

CpG islands (CGIs) are genomic regions that contain a high content of CpG repeats. They are usually located within or close to the promoters (Larsen et al., 1992). Unlike other CpG sites, CpG repeats within CGIs are usually unmethylated, and this unmethylation state is associated with the active transcriptional activity. Therefore, we examined the distributions of 5mC and 5hmC in CGIs. As shown in Figures 2A, B as well as Supplementary Figures S2A, B, OSCC group has a slightly higher level of 5mC than NAT group, but similar 5hmC contents are seen in both groups. In CGI surrounding areas, including CGI shores (regions up to 2 kb upstream and downstream from CGIs) and CGI shelves (regions 2–4 kb upstream and downstream from CGIs) (Rechache et al., 2012), both 5mC and 5hmC are similarly decreased in OSCC group in comparison to NAT group.

Next, we investigated the distributions of 5mC and 5hmC in individual genomic regions, including gene bodies, 5 kb upstream transcription start sites (TSSs), and 5 kb downstream of transcription termination sites (TTSs). Our results show that, relative to NAT group, the level of 5mC is reduced in all these regions in OSCC group (Figure 2C; Supplementary Figure S2C), and similarly, the amount of 5hmC is also decreased in all these regions in OSCC group (Figure 2D; Supplementary Figure S2D). Within gene bodies and at specific regions (such as promoter, exon, intron, UTRs, et al.), decreased 5mC- and 5hmC-contents are consistently seen in OSCC in contrast with NAT samples (Figures 2E, F; Supplementary Figures S2E, F). Therefore, there might be an overall reduction of 5mC and 5hmC in various genomic regions in OSCC tissues.

### 2.3 Identification of promoter DMRs and DhMRs

**FIGURE 3:**
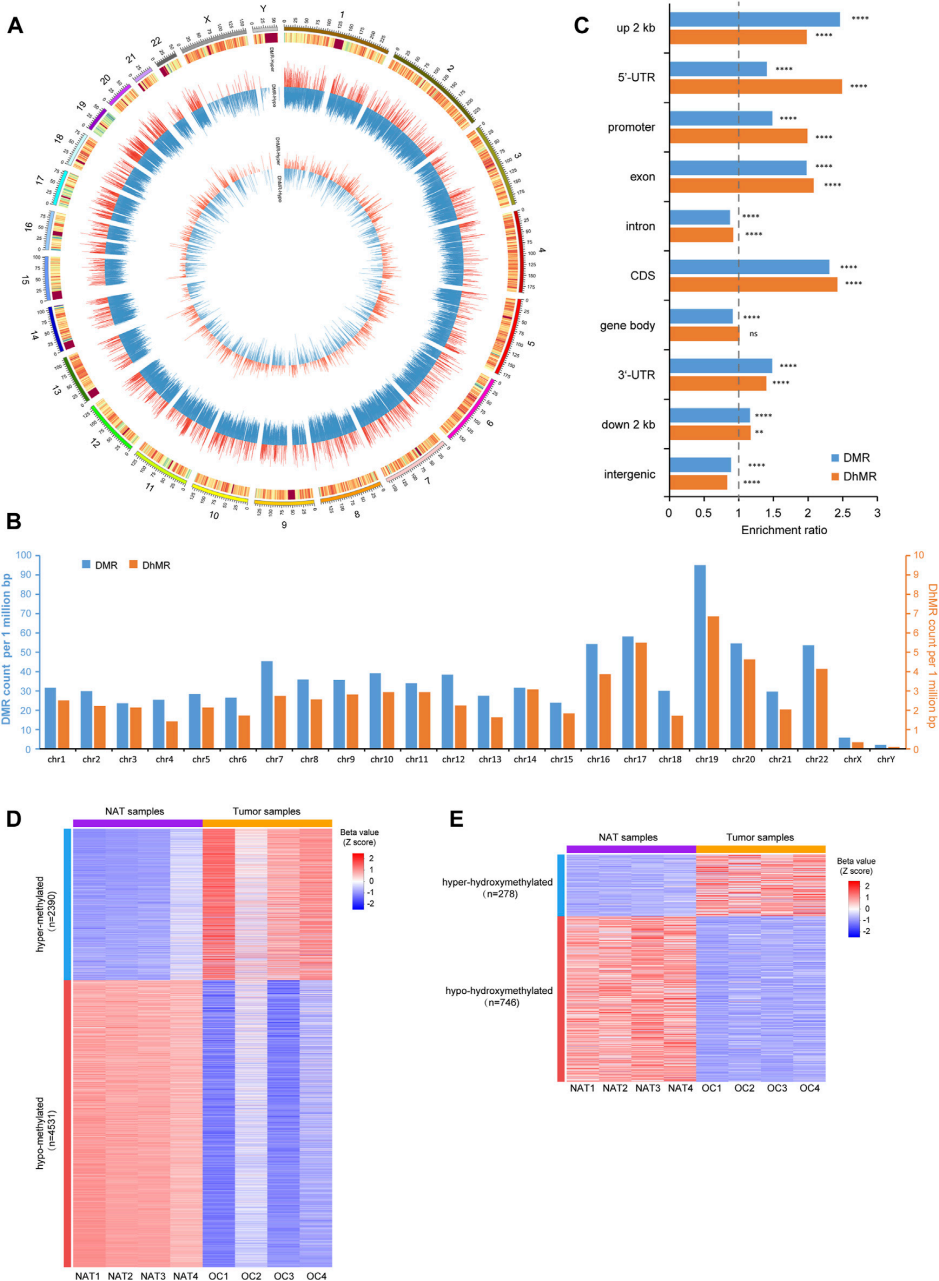
Identification of p-DMRs and p-DhMRs **(A)**. Circos plot shows the genomic distribution of DMRs and DhMRs across all chromosomes. Each individual bar within the middle and inner circles represents either a DMR or a DhMR, respectively. The height of each bar indicates the extent of methylation difference (Δβ value), while a red bar denotes hypermethylated and a blue bar denotes hypomethylated, respectively. **(B)**. Bar plot shows the distribution of DMRs and DhMRs in each chromosome. The *Y*-axis indicates the DMRs/DhMRs count per 1 million base pairs. **(C)**. Bar plot shows the genewise distribution of DMRs and DhMRs in each genomic feature. The *Y*-axis indicates specific genomic regions and the *X*-axis indicates the enrichment ratio of DMRs/DhMRs. **(D)**. Heatmap represents the level of p-DMRs (Z score of β value) in NAT and OSCC samples, including 2,390 hyper-methylated and 4,531 hypo-methylated p-DMRs. **(E)**. Heatmap represents the level of p-DhMRs (Z score of β value) in NAT and tumor samples, including 278 hyper-hydroxymethylated and 746 hypo-hydroxymethylated p-DhMRs. NAT: normal adjacent tissue. OSCC: oral squamous cell carcinoma. DMRs: differential methylation regions. DhMRs: differential hydroxymethylation regions. p-DMRs: DMRs localized at the promoters. p-DhMRs: DhMRs localized at the promoters. **p < .01. ****p < .0001.

Next, we identified differentially methylated regions (DMRs) and differentially hydroxymethylated regions (DhMRs) comprising multiple consecutive CpG sites. Since the overall difference of 5mC between OSCC and NAT groups is more remarkable than that of 5hmC, we adjusted the stringency of cutoff criteria accordingly to avoid over-selection of DMRs against DhMRs. A total of 104,109 DMRs and 7,710 DhMRs were identified by comparing 5mC- and 5hmC-profiles between OSCC and NAT groups (Supplementary Table S1, Supplementary Table S2). Genomewise, these DMRs and DhMRs are distributed across all chromosomes with preference for autosomes (Figures 3A, B). Genewise, DMRs and DhMRs spread across various genomic features similarly, with more preference in promoters, UTRs, and exons and less, in introns and intergenic regions (Figure 3C).

Considering the crucial role of DNA modification at the promoters in regulating gene expression, we further selected out differentially methylated promoter regions (p-DMRs) and differentially hydroxymethylated promoter regions (p-DhMRs). A total of 6,921 p-DMRs and 1,024 p-DhMRs were identified between two conditions (Supplementary Table S3, Supplementary Table S4). Within these p-DMRs and p-DhMRs, 2,390 are hyper-methylated (up-5mC) and 4,531 are hypo-methylated (down-5mC); 278 are hyper-hydroxymethylated (up-5hmC) and 746 are hypo-hydroxymethylated (down-5hmC). Heatmap of these p-DMRs and p-DhMRs are shown in Figures 3D, E, respectively.

### 2.4 Integrative analyses between methylation, hydroxymethylation, and gene expression

To assess the relationship between cytosine modifications and gene expression in the context of OSCC, we integrated the RNA-sequencing data previously obtained from the same samples that are used for WGBS and oxWGBS in this study (Wan et al., 2021). Only genes expressed in at least 25% of all samples were selected out for integrated multi-omics analyses between gene expression and 5mC- or 5hmC-modifications.

**FIGURE 4:**
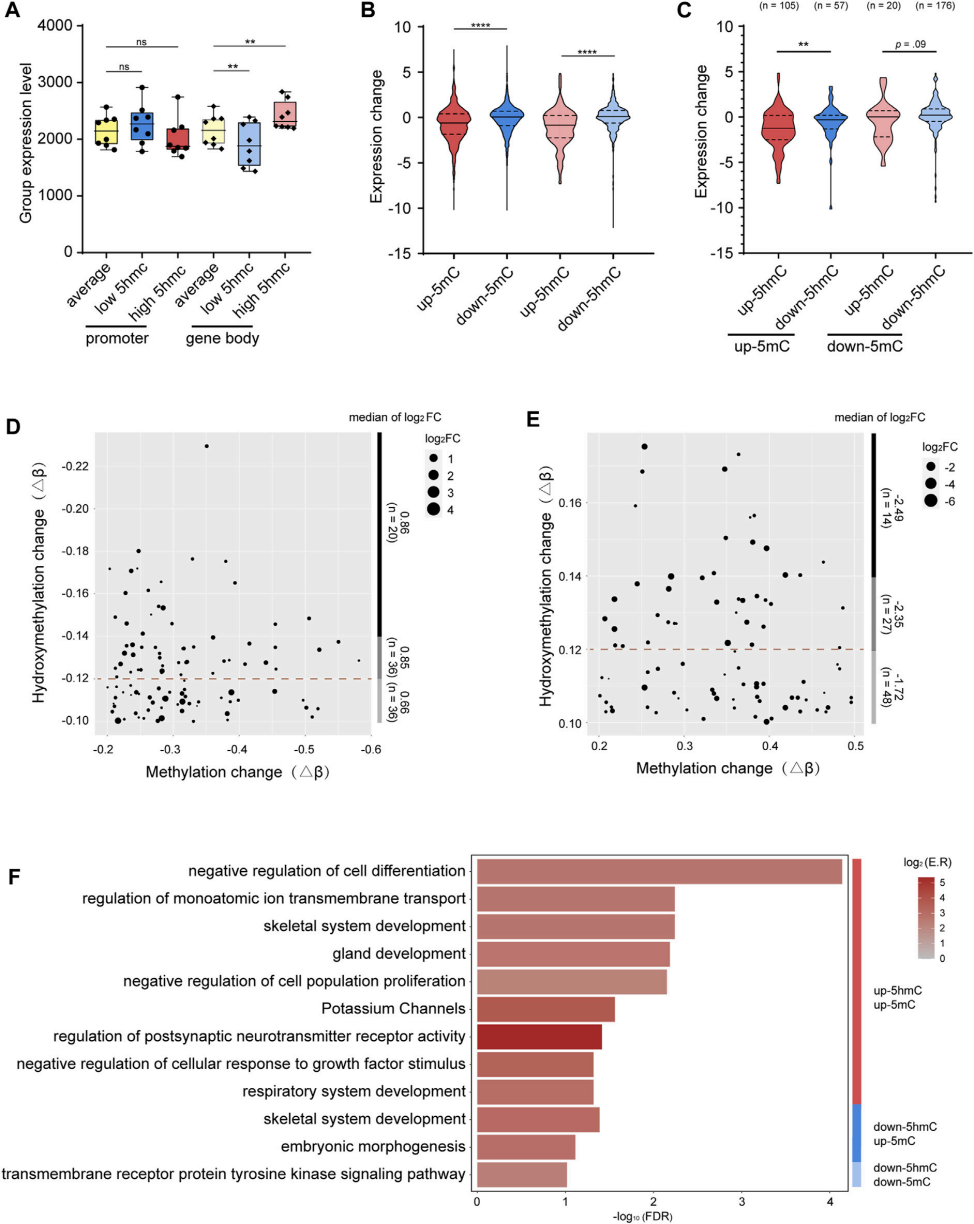
Integrative analysis between 5mC, 5hmC, and gene expression **(A)**. Grouped expression level (mean normalized count of each group) of genes with average- (yellow), low- (blue), and high-levels (red) of 5hmC at the promoters (standard color) or gene bodies (lighter color). **(B)**. Grouped expression changes of genes with hyper-methylated p-DMR (red), hypo-methylated p-DMR (blue), hyper-hydroxymethylated p-DhMR (lighter red), and hypo-hydroxymethylated p-DhMR (lighter blue). **(C)**. Grouped expression changes of genes with various combinations of 5mC-and 5hmC-alterations at the promoters. Up-5mC/up-5hmC (red), up-5mC/down-5hmC (blue), down-5mC/up-5hmC (lighter red), and down-5mC/down-5hmC (lighter blue). **(D)**. Dot plot shows the coordination between the level of gene upregulation and the level of down-5hmC among genes with unidirectional down-5mC/down-5hmC at the promoters. **(E)**. Dot plot shows the coordination between the level of gene downregulation and the level of up-5hmC among genes with unidirectional up-5mC/up-5hmC at the promoters. **(F)**. Bar plot shows functional clusters of genes with the unidirectional or bidirectional alterations of 5mC and 5hmC at promoter regions. -log_10_ (FDR) means -log_10_ transformed FDR, color bar indicates log_2_ (enrichment ratio) with red color representing higher expression levels and grey color representing lower expression levels. p-DMRs: DMRs localized at the promoters. p-DhMRs: DhMRs localized at the promoters. Up-5mC: hyper-methylated. Down-5mC: hypo-methylated. Up-5hmC: hyper-hydroxymethylated. Down-5hmC: hypo-hydroxymethylated. Log_2_FC: log_2_ fold change. **p < .01. ****p < .0001.

We first asked whether the level of gene-body 5hmC correlates with the level of gene expression. To minimize the interference from the promoter 5mC, only genes with low 5mC at promoter regions (average β value < 0.1) were included in this analysis. Indeed, we found that genes with higher level of gene-body 5hmC are more expressed, while genes with lower level of gene body 5hmC are less expressed (Figure 4A). These data are consistent with previous reports showing 5hmC modification at the gene body is positively correlated with gene expression (Ficz et al., 2011; Wu and Zhang, 2017). However, with genes having low 5mC at their promoters, we saw no significant correlation between the levels of promoter 5hmC and gene expression (Figure 4A).

Next, we investigated the potential effect of the promoter 5mC and 5hmC on gene expression in the context of OSCC. After screening out genes expressed in less than 25% of all the samples, there are in total 3,568 genes with p-DMRs (p-DMGs), 1,156 promoter up-5mC and 2,412 promoter down-5mC, and 764 genes with p-DhMRs (p-DhMGs), 165 promoter up-5hmC and 599 promoter down-5hmC. As shown in Figure 4B, genes with up-5mC at their promoters (*n* = 1156) are significantly less expressed than genes with down-5mC at their promoters (*n* = 2412), reflecting the negative correlation between promoter 5mC and gene expression that is consistent with previous reports (Luo et al., 2018). Interestingly, a negative correlation was also noticed between promoter 5hmC and gene expression, i.e., genes with up-5hmC at the promoters (*n* = 165) have less mRNA levels than genes with down-5hmC (*n* = 599) (Figure 4B). Our data suggest that 5hmC at the promoters could have different effect on gene expression than 5hmC in other genomic regions.

To understand the potential interaction between promoter 5mC and promoter 5hmC and its effect on gene expression in OSCC, from the above mentioned 3,568 genes, we further selected out the subset of 358 genes that bear both p-DMR and p-DhMR (Supplementary Table S5). Of the 162 p-DMGs containing up-5mC at their promoters, 64.81% also contain up-5hmC (105 up-5hmC/up-5mC in 162 up-5mC p-DMGs). Similarly, among the 196 p-DMGs having down-5mC at their promoters, 89.80% also have down-5hmC (176 down-5hmC/down-5mC in 196 down-5mC p-DMGs). We hence name such compound, unidirectional modifications with both 5mC and 5hmC (up-5mC/up-5hmC and down-5mC/down-5hmC) “unidirectional modification.” Accordingly, we name the other two patterns (up-5mC/down-5hmC and down-5mC/up-5hmC) “bidirectional modification.” As shown in Supplementary Figure S3, 73.33% (77 out of 105) genes bearing unidirectional modification with up-5mC/up-5hmC show reduced gene expression; 57.39% (101 out of 176) genes bearing unidirectional modification with down-5mC/down-5hmC have increased mRNA levels. Moreover, within these p-DMGs and p-DhMGs, we found that the reduction of gene expression is significantly bigger in genes carrying unidirectional modification with up-5mC/up-5hmC than in genes carrying bidirectional modification with up-5mC/down-5hmC (*p* = .003); likewise, genes bearing unidirectional modification with down-5mC/down-5hmC present a larger increase of their mRNA levels than genes carrying bidirectional modification with down-5mC/up-5hmC (*p* = .09, Figure 4C).

When we stratified above-mentioned p-DMGs by the level of 5hmC alteration (up-5mC p-DMGs with high, medium, or low 5hmC-enrichment; down-5mC p-DMGs with high, medium, or low 5hmC-reduction), we noticed that the level of 5hmC alteration is in proportion to the degree of the change of gene expression: larger 5hmC-reduction is associated with greater upregulation of gene expression (log_2_FC = 0.66 in top 1/3 5hmC-reduction and log_2_FC = 0.86 in bottom 1/3 5hmC-reduction), and bigger 5hmC-enrichment is related to stronger downregulation of gene expression (log_2_FC = −2.49 in top 1/3 5hmC-enhancement and log_2_FC = −1.72 in bottom 1/3 5hmC-enhancement, Figures 4D, E). Our observation suggests a possibility that, at the promoters, 5hmC might facilitate 5mC’s function in repressing gene expression. As a result, we asked whether these unidirectional modified genes are likely to be functionally involved in tumor pathology.

### 2.5 Functional analysis of differentially expressed genes with promoter-unidirectional-modification with 5mC and 5hmC in OSCC

To assess the functional relevance of the genes bearing promoter-unidirectional-modification with 5mC and 5hmC to the OSCC pathology, we performed pathway analyses of the sets of genes with various combinations of 5mC- and 5hmC-alterations at the promoters, including up-5mC/up-5hmC, down-5mC/down-5hmC, up-5mC/down-5hmC, and down-5mC/up-5hmC. Although the effects of epigenetic modification on biological functions are complicated, the enrichment result shows that genes with promoter-unidirectional-modifications are more enriched in pathways directly associated with tumorigenesis (for example, negative regulation of cell differentiation, negative regulation of cell population proliferation, and negative regulation of cellular response to growth factor stimulus are enriched in the gene set with up-5mC/up-5hmC; transmembrane receptor protein tyrosine kinase signaling pathway are enriched in the gene set with down-5mC/down-5hmC) (Figure 4F; Supplementary Figure S4). In contrast, genes with bidirectional modifications at the promoters are enriched in pathways less directly associated with initial oncogenesis, such as skeletal system development and embryonic morphogenesis (Figure 4F; Supplementary Figure S4).

**FIGURE 5:**
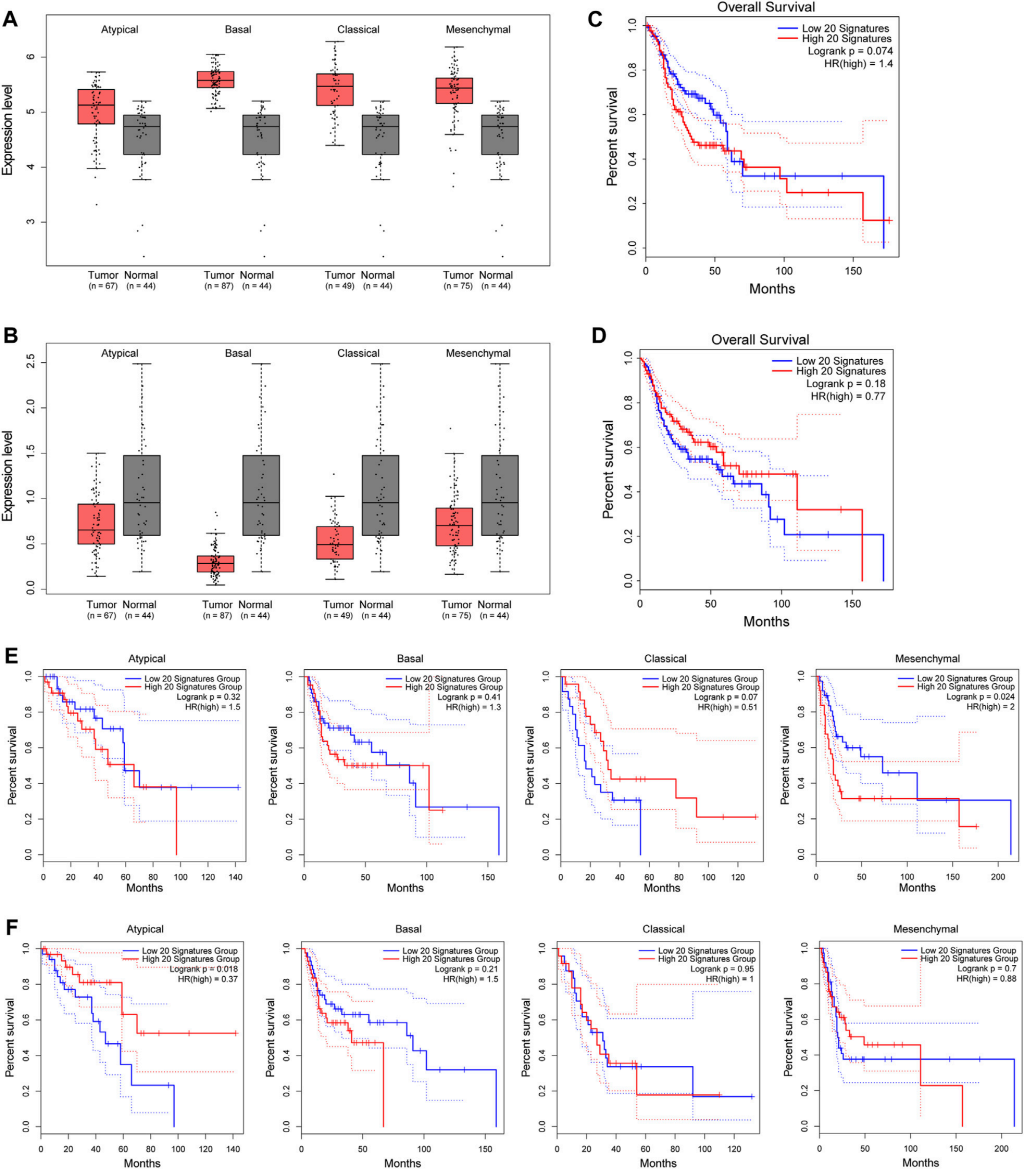
Clinical relevance of genes with unidirectional modification with promoter 5mC and 5hmC to HNSC. **(A–B)**. Box plots show the grouped expression levels of the top 20 upregulated genes with down-5mC/down-5hmC at their promoters **(A)** and the top 20 downregulated genes with up-5mC/up-5hmC at their promoters **(B)** in four subtypes of HNSC tumor and matched normal tissues (TCGA “solid tissue normal” samples from HNSC patients). The group expression level is defined as the mean log2 (TPM + 1) of each group. **(C–D)**. Kaplan-Meier plots show the OS curves of HNSC patients with a low or high signature score of the above-mentioned top 20 upregulated genes **(C)** and of the above-mentioned top 20 downregulated gene signatures **(D)**. **(E–F)**. Kaplan-Meier plots show the OS curves of each subtype of HNSC patients with a low or high signature score of the above-mentioned top 20 upregulated genes **(E)** and of the above-mentioned top 20 downregulated genes **(F)** in four pathological subtypes of HNSC. Blue line: low signature score. Red line: high signature score. HNSC: head and neck squamous cell carcinoma. TPM: transcript per million. OS: overall survival.

Next, we selected out the top 20 upregulated genes with down-5mC/down-5hmC at their promoters (top 20 down-5mC/down-5hmC) and the top 20 downregulated genes with up-5mC/up-5hmC at their promoters (top 20 up-5mC/up-5hmC), and their grouped expression signatures were further validated in TCGA HNSC dataset, of which majority of HNSC cases are OSCCs. As shown in Figures 5A, B, the grouped expression levels of top 20 down-5mC/down-5hmC and top 20 up-5mC/up-5hmC are not significantly changed in all four pathological subtypes of HNSC. However, when examining the association between the grouped expression level of the top 20 down-5mC/down-5hmC or of the top 20 up-5mC/up-5hmC genes and the OS of HNSC patients, we noticed their signature scores tend to correlate with the certain HNSC clinical outcomes. High expression level of grouped top 20 down-5mC/down-5hmC genes tends to be associated with shorter OS (Figure 5C), with statistical significance in mesenchymal type of HNSC (Figure 5E); while low expression of grouped top 20 up-5mC/up-5hmC genes tends to relate to shorter OS (Figure 5D), with statistical significance in atypical type of HNSC (Figure 5F). Therefore, our bioinformatic analysis suggests that genes bearing promoter-unidirectional-modification with 5-mC and 5-hmC might have clinical relevance in OSCC.

## 3 Discussion

Recent studies have suggested the alterations of 5hmC, either global or regional, are involved in cancers, needless to say that its precursor, 5mC, is one of the most well-known epigenetic features in cancer biology. OSCC is the sixth most common malignancy worldwide and has a poor clinical outcome. Understanding the molecular changes that lead to the onset and development of OSCC is essential to improve the clinical management of this disease. Previous studies have revealed a global depletion of 5mC and 5hmC in OSCC using immunohistochemical staining (Piyathilake et al., 2005; Wang et al., 2017; Cuevas-Nunez et al., 2018). Array-based methods have been used to analyze the genome-wide distribution of 5mC and identify relevant biomarkers in OSCC (Pickering et al., 2013; Towle et al., 2013; Langevin et al., 2014; Basu et al., 2017), while single-base resolution analysis of 5mCs in OSCC is still lacking. For 5hmC, Liu and colleagues reported the first genome-wide 5hmC profile in HNSC using hydroxymethylated DNA immunoprecipitation and sequencing (hMeDIP-seq) (Liu et al., 2020). However, this approach has relatively low resolution and cannot quantitatively determine the abundance of 5hmC at individual modification site. Therefore, a precise, genome-wide 5hmC profiling in OSCC at single-nucleotide resolution is still unavailable.

oxWGBS, together with WGBS, is a genome-wide, single-base sequencing technique that can distinguish between 5mC and 5hmC via the highly selective chemical oxidation of 5hmC to 5 fC (Booth et al., 2012). It first uses potassium perruthenate (KRuO4) oxidation treatment to convert 5hmC to 5 fC, then converts 5 fC to uracil in the subsequent sulfite-treatment and reads uracil as thymine in the final sequencing step (Booth et al., 2013). Since 5mC does not undergo chemical oxidation, it will be detected as C during oxWGBS. By comparing the data from WGBS (which indistinguishably identifies both 5hmC and 5mC) and data from oxWGBS (which only identifies 5mC), the site and level of 5hmC can be precisely mapped and quantified. In this study, we carried out these sequencings to map the 5mC- and 5hmC-contents in paired OSCC and NAT samples.

Compared with NAT group, OSCC group has a similar level of 5hmC and a lower level of 5mC across the entire genome. In CpG sites, however, the ratio of CpG sites containing 5mC and the ratio of those containing 5hmC are both lower in OSCC than in NAT samples. In CGIs, more 5mC content, but not 5hmC, are seen in OSCC, relative to NAT; in CGI adjacent areas, including CGI shores and CGI shelves, both 5mC and 5hmC are less abundant in OSCC than those in NAT. All these data suggest that, at the whole genome level, 5mC and 5hmC might have different distribution patterns between cancerous and normal tissues. Taking a deeper look at CpG sites in various functional genomic regions, including 5′-UTR, promoter, gene body, 3′-UTR, and the flanking sequences around TSSs/TTSs, we noticed that OSCC samples have reduced abundance of both 5mC and 5hmC in all these genomic regions when compared to NATs. Thus, both 5mC and 5hmC are downregulated in these areas that are important for gene expression. To the best of our knowledge, we are the first to report that there might be similar overall reductions of 5mC and 5hmC in various genomic regions in OSCC, using parallel WGBS and oxWGBS.

Increasing evidence has suggested that gene-body 5hmC is positively correlated with the expression of the marked gene (Ficz et al., 2011; Wu and Zhang, 2017), a correlation we also observed in this study in the context of OSCC. Several mechanistic hypotheses, such as 5hmC’s repelling of 5mC-binding proteins or 5hmC’s recruiting of 5hmC-binding proteins, have also been proposed for this positive regulation of gene expression by gene-body 5hmC (Jin et al., 2010; Yildirim et al., 2011; Spruijt et al., 2013; Wu and Zhang, 2017). Unlike gene-body 5hmC, promoter 5hmC has been reported playing more complicated effect on gene expression, with positive regulations in certain situations (Ficz et al., 2011; Wernig-Zorc et al., 2019) but negative regulations in others (Stroud et al., 2011; Wu et al., 2011; Greco et al., 2016). In our study, a significant enrichment of DhMRs was observed at the promoter regions, and a negative correlation was noticed between the promoter 5hmC and gene expression in OSCC. However, after excluding the potential interference from the promoter 5mC, no obvious correlation could be drawn between them. Therefore, our data support a complicated effect of promoter 5hmC on gene expression. Moreover, the difference between the regulatory effects by promoter and gene-body 5hmC raises the safety concern of developing anti-cancer medicine that targets global 5hmC-alteration.

To understand the functional profile of 5mC and 5hmC at the promoters, we first identified a total of 6,921 p-DMRs and 1,024 p-DhMRs between OSCC and NAT groups. Second, we examined the relationship between the mRNA level of and the differentiated expressed amount of 5mC or 5hmC in each gene. Our analysis revealed that genes with compound, unidirectional alteration with 5mC and 5hmC at their promoters presented bigger changes in their mRNA levels than those with promoter-bidirectional-modification with 5mC and 5hmC. Our observation raises the possibility that, at the promoters, 5hmC might facilitate 5mC’s role as a gene expression repressor in OSCC. Indeed, we noticed that differentially expressed genes having promoter-unidirectional-modification with 5mC and 5hmC not only are directly associated with signaling pathways of tumorigenesis but also present clinical relevance—the grouped expression levels of top 20 up-5mC/up-5hmC genes and top 20 down-5mC/down-5hmC genes tend to associate with the OS of HNSC, with statistical significance in mesenchymal and atypical subtypes. However, more studies are needed to elucidate whether this association indicates a direct regulation of gene expression by 5mC and 5hmC at the promoters, or a bypass phenomenon associated with altered DNA demethylation in hyper-proliferative cells. Our study, although limited by relatively small sample size, suggests that, in OSCC, unidirectional alterations of 5mC and 5hmC at the promoters may mark the dynamically regulated genes that play important roles in the tumorigenesis. Furthermore, differentially regulated genes carrying promoter-unidirectional-modification with 5mC and 5hmC are likely to be relevant to the disease progression in OSCC. Further investigations with a larger sample size and more in-depth multi-omic analyses are needed to shed more light on this topic.

## 4 Conclusion

In summary, this study provides the first genome-wide, single-base-resolution, parallel mapping of 5mC and 5hmC in OSCC. Using an integrated multi-omics approach, we observed an enhanced expression change with genes carrying unidirectional modification with 5mC and 5hmC at the promoters in OSCC samples. In addition, suchlike differentially regulated genes bearing promoter-unidirectional-modification with 5mC and 5hmC might have functional relevance to the clinical outcome of OSCC.

## 5 Materials and methods

### 5.1 Tumor biospecimen collection and RNA extraction

Four pairs of OSCC tissue and NAT (normal adjacent epithelial tissue 2 cm away from paired tumor), in a total of eight samples, were collected from patients who underwent surgical resection and had received no prior treatment for the disease at Xiangya Hospital of Central South University (CSU), Changsha, Hunan, China. Patients’ clinical characteristics are presented in Table 4. The samples were named as OC or NAT (stands for OSCC and NAT, respectively), followed by patient number (for example, OC1 and NAT1 from patient 1, OC2 and NAT2 from patient 2, etc.). All samples were collected in accordance with the guidelines issued by the Ethics Committee of the School of Life Sciences, CSU. Written informed consent was obtained from all participants in this study.

**TABLE 4:** Patients’ clinical characteristics.

	Age (years)	Gender	Cancer site	Degree of differentiation	Stage
Patient 1 (Samples OC1 and NAT1)	62	Female	Inner cheek	Moderately-differentiated	III
Patient 2 (Samples OC2 and NAT2)	48	Male	Gingiva	Well-differentiated	II
Patient 3 (Samples OC3 and NAT3)	63	Male	Inner cheek	Moderately-differentiated	III
Patient 4 (Samples OC4 and NAT4)	71	Male	Gingiva	Well-differentiated	II

After surgical removal, all samples were immediately put into a CO_2_-independent medium (Cat #18045-088, Gibco, USA) and transferred to the laboratory on ice. About 30 mg of each pure tumor and NAT sample was further dissected and used for extraction of DNA and total RNA following the manufacturer’s instruction of the AllPrep DNA/RNA/miRNA Universal Kit (Cat #80224, Qiagen, USA). All biospecimens were pathologically reviewed and confirmed by two independent and certified pathologists.

### 5.2 Bisulfite sequencing (BS-seq) and oxidative bisulfite sequencing (oxBS-seq) library preparation and sequencing

BS-seq and oxBS-seq were performed as previously described (Booth et al., 2013). 2 μg genomic DNA mixed with unmethylated lambda DNA was fragmented to an average size of 250 bp with S220 Focused-ultrasonicator (Covaris, USA). The randomly fragmented DNA was subsequently subjected to constructed libraries using TrueMethyl oxBS Module (Cat #0414, Tecan, USA). Briefly, the fragmented DNA was firstly treated with a combination of T4 DNA polymerase, Klenow fragment, and T4 polynucleotide kinase; then the blunted DNA fragments were added with sequencing spike-in control DNA duplex (containing SQ6hmC, SQ3hmC, SQ1hmC, SQC, SQmC, and SQfC) and were subsequently 3′ adenylated using Klenow fragment and ligated to 5mC adaptors using T4 DNA Ligase; the ligated products were treated either with 1 µL Oxidant Solution for oxBS-seq libraries or 1 μL Ultra-Pure Water for BS-seq libraries, followed by bisulfite conversion using Bisulfite Reagent. After PCR amplification using barcode-tagged primers, all 16 libraries (eight oxBS-seq libraries and eight corresponding BS-seq libraries) were pooled and sequenced on a HiSeq 3,000 sequencer (Illumina, USA) using 150 bp paired-end model.

### 5.3 BS-seq and oxBS-seq data processing

First, adapters were removed, and low-quality reads were filtered out by Trim Galore (version 0.5.0). Then clean reads were aligned against the human reference genome (hg38) by BSMAP (version 2.73). After alignment, methylation measurements for each CpG site (β value) were extracted from aligned reads using methratio.py (a Python-based script embedded in BSMAP). The hydroxymethylation measurements were obtained by running the MLML algorithm in the MethPipe package (Qu et al., 2013) (version 4.1.1) over BS-seq and oxBS-seq data. Mapped CpG sites with coverage no less than 10-fold were included for further analysis.

Groupwise differentially methylated regions (DMRs) and differentially hydroxymethylated regions (DhMRs) were analyzed by Metilene (version 0.2.6), a circular binary segmentation algorithm, using the following parameters: DMR was defined as regions with |Δβ| > 0.2, number of CpG > 10 per region, CpG density in region > 2%, and FDR < 0.001; DhMR was defined as regions with |Δβ| > 0.1, number of CpG ≥ 5 per region, and FDR < 0.05.

### 5.4 Distribution analysis of 5mC and 5hmC

Genomic annotations of the hg38 were obtained from the UCSC Genome Browser Database (https://hgdownload.soe.ucsc.edu/). Genomic regions annotated as 5′-untranslated region (5′-UTR), promoter, gene body, exon, intron, and 3′-untranslated region (3′-UTR) were extracted and divided into 30 bins. The average 5mC and 5hmC levels in each bin were calculated and plotted.

The enrichment of DMRs or DhMRs in each annotated region was calculated as the observed frequency of genomic region over the expected frequency. The expected frequency represents the number of significant DMRs or DhMRs over the total number of DMRs or DhMRs across the whole genome. The observed frequency of genomic region represents the number of significant DMRs or DhMRs over the total number of DMRs or DhMRs in this genomic region. Fisher’s exact test were applied, and Benjamini-Hochberg adjusted *p-*values were calculated to establish statistical significance.

### 5.5 Transcriptome data processing and correlation analysis

Transcriptome data of all samples were previously obtained through RNA sequencing (Wan et al., 2021). For short, 1 μg of total RNA from each sample was depleted for rRNA using Ribo-Zero rRNA Removal Kit (Cat #RZH1046, EPICENTRE, USA), followed by the construction of sequencing library using the TruSeq RNA Library Prep Kit v2 (Cat #RS-122, Illumina, USA). Pooled RNA libraries were sequenced on a HiSeq3000 using 150 bp paired-end model. Raw data have been deposited into the NCBI GEO database (https://www.ncbi.nlm.nih.gov/geo/, GSE186775).

To analyze 5hmC’s regulatory effect on gene expression, we only selected genes with low levels of promoter 5mC (average β value < 0.1) to minimize the influence from promoter 5mCs, then divided them into promoter high-5hmC, promoter low-5hmC, gene-body high-5hmC, and gene-body low-5hmC according to their median values across all samples, and calculated the grouped expression level of genes in each group.

To analyze the regulatory effect by promoter modification of 5mC and 5hmC, the DMRs and DhMRs localized at the promoters (p-DMRs and p-DhMRs) were selected for further analysis. The expression levels of genes with p-DMRs and p-DhMRs (p-DMGs and p-DhMGs) were extracted from transcriptome data if their transcriptions were detected in at least 25% of all samples combined from both NAT and OSCC groups, based on previously described method ^[50]^ . According to the alteration pattern of 5mC and 5hmC at the promoters, these p-DMGs and p-DhMGs were divided into four groups, and the expression levels of grouped genes were compared using Mann–Whitney U test with exact *p-*values.

### 5.6 Functional enrichment analysis

Functional enrichment analyses, including Gene Ontology (GO) Biological Processes and Reactome Gene Sets, were performed using the Metascape web server (https://metascape.org/) with default parameters: a minimal overlap of 3, a minimal enrichment ratio of 1.5, and a maximum *p-*value of .01, as previously described (Wan et al., 2021).

### 5.7 Analysis of genes with unidirectional alternations with 5mC and 5hmC

First, genes with unidirectional alterations with 5mC and 5hmC at the promoters were stratified into three groups by their level of 5hmC alteration: high 5hmC alteration (|Δβ| > 0.14), medium 5hmC alteration (0.14 ≥ |Δβ| > 0.12), and low 5hmC alteration (0.12 ≥ |Δβ| > 0.10). Then, the median log_2_ fold change (log_2_FC) of each group was calculated using the formula log_2_FC = log_2_ (grouped expression of OSCC)—log_2_ (grouped expression of NAT). The top 20 upregulated genes with at least a medium reduction of 5hmC and the top 20 downregulated genes with at least a medium enrichment of 5hmC were further selected out and analyzed for their functional relevance to OSCC.

### 5.8 Validation of top differentially expressed genes with unidirectional modification with 5mC and 5hmC

Clinical relevance of grouped top 20 upregulated genes and top 20 downregulated genes was evaluated in four different subtypes (atypical-, basal-, classical-, and mesenchymal-subtype) of head and neck squamous cell carcinoma (HNSC) using the GEPIA (Gene Expression Profiling Interactive Analysis) web server (Tang et al., 2019). The expression level of these grouped genes was first validated in each of these four subtypes of HNSC. The group expression level is defined as the mean log_2_ (TPM + 1) of the genes in each group, and TPM stands for transcript per million. The overall survival (OS) of HNSC cases with high (>75th percentile) and low (<25th percentile) expression levels of these grouped genes were plotted using the Kaplan-Meier (KM) curve. Similar OS curves were plotted with each subtype of HNSC cases but with slightly different settings (high means > 50th percentile, and low means < 50th percentile).

### 5.9 Visualization

The Circos plot was generated by the Circos software (version 0.69, http://circos.ca/). The heatmap was plotted with the “heatmap.2” function of the “gplots” package provided in the R environment (version 3.1.3, https://github.com/talgalili/gplots). Other graphs including the violin plot, dot plot, and bar plot were visualized using the ggplot2 package in R environment (version 3.3.6, https://ggplot2.tidyverse.org/).

## Data Availability

The datasets presented in this study can be found in online repositories. The names of the repository/repositories and accession number(s) can be found in the article/Supplementary Material.

## Supplementary material

SUPPLEMENTARY FIGURE S1 Cluster dendrogram of DNA methylation and hydroxymethylation in OSCC and NAT samples. **(A)**. Cluster dendrogram shows DNA methylation in OSCC and NAT samples. **(B)**. Cluster dendrogram shows DNA hydroxymethylation in OSCC and NAT samples. OSCC: oral squamous cell carcinoma. NAT: normal adjacent tissue.

SUPPLEMENTARY FIGURE S2 Distribution of 5mC and 5hmC in gene regulatory elements for each individual sample. **(A)**. Methylation rate at CGI, CGI ± 2 kb region, and CGI ± 4 kb region for each sample. **(B)**. Hydroxymethylation rate at CGI, CGI ± 2 kb region, and CGI ± 4 kb region for each sample. **(C)**. Methylation rate at gene body, 5 kb upstream TSSs, and 5 kb downstream TTSs for each sample. **(D)**. Hydroxymethylation rate at gene body, 5 kb upstream TSSs, and 5 kb downstream TTSs for each sample. **(E)**. Methylation rate of 5mC at 2 kb upstream 5′-UTR, 5′-UTR, promoter, gene body, exon, intron, 3′-UTR, 2 kb downstream 3′-UTR for each sample. **(F)**. Hydroxymethylation rate at 2 kb upstream 5′-UTR, 5′-UTR, promoter, gene body, exon, intron, 3′-UTR, 2 kb downstream 3′-UTR for each sample. Blue- and red-colored areas with the mean line represents 95% confidence intervals of NAT and tumor samples, respectively. CGIs: CpG islands. TSSs: transcription start sites. TTSs: transcription termination sites. 5′-UTR: 5′-untranslated region. 3′-UTR: 3′-untranslated region.

SUPPLEMENTARY FIGURE S3 Association between the change of gene expression and the pattern of 5mC- and 5hmC-modification at the promoters. Grouped expression changes of upregulated genes (solid line) or downregulated genes (dashed line) carrying various combinations of 5mC- and 5hmC-alterations at the promoters. Up-5mC/up-5hmC (red), up-5mC/down-5hmC (blue), down-5mC/up-5hmC (lighter red), and down-5mC/down-5hmC (lighter blue). Up-5mC: hyper-methylated. Down-5mC: hypo-methylated. Up-5hmC: hyper-hydroxymethylated. Down-5hmC: hypo-hydroxymethylated.

SUPPLEMENTARY FIGURE S4 Functional analysis of the unidirectional or bidirectional alterations of 5mC and 5hmC at promoter regions. Bar plot shows functional clusters of genes with the unidirectional or bidirectional alterations of 5mC and 5hmC at promoter regions. -log10 (p-value) means -log10 transformed p-value, color bar indicates log2 (enrichment ratio) with red color representing higher expression levels and grey color representing lower expression levels. Up-5mC: hyper-methylated. Down-5mC: hypo-methylated. Up-5hmC: hyper-hydroxymethylated. Down-5hmC: hypo-hydroxymethylated.

## References

[B1] Azizgolshani N. Petersen C. L. Chen Y. Levy J. J. Salas L. A. Perreard L. (2021). DNA 5-hydroxymethylcytosine in pediatric central nervous system tumors may impact tumor classification and is a positive prognostic marker. Clin. Epigenetics 13 (1), 176. 10.1186/s13148-021-01156-9 34538273 PMC8451154

[B2] Basu B. Chakraborty J. Chandra A. Katarkar A. Baldevbhai J. R. K. Dhar Chowdhury D. (2017). Genome-wide DNA methylation profile identified a unique set of differentially methylated immune genes in oral squamous cell carcinoma patients in India. Clin. Epigenetics 9, 13. 10.1186/s13148-017-0314-x 28174608 PMC5292006

[B3] Booth M. J. Branco M. R. Ficz G. Oxley D. Krueger F. Reik W. (2012). Quantitative sequencing of 5-methylcytosine and 5-hydroxymethylcytosine at single-base resolution. Science 336 (6083), 934–937. 10.1126/science.1220671 22539555

[B4] Booth M. J. Ost T. W. Beraldi D. Bell N. M. Branco M. R. Reik W. (2013). Oxidative bisulfite sequencing of 5-methylcytosine and 5-hydroxymethylcytosine. Nat. Protoc. 8 (10), 1841–1851. 10.1038/nprot.2013.115 24008380 PMC3919000

[B5] Choy J. S. Wei S. Lee J. Y. Tan S. Chu S. Lee T. H. (2010). DNA methylation increases nucleosome compaction and rigidity. J. Am. Chem. Soc. 132 (6), 1782–1783. 10.1021/ja910264z 20095602 PMC4167393

[B50] Cramer J. D. Burtness B. Le Q. T. Ferris R. L. (2019). The changing therapeutic landscape of head and neck cancer. Nat. Rev. Clin. Oncol. 16 (11), 669–683. 31189965 10.1038/s41571-019-0227-z

[B6] Cuevas-Nunez M. C. Gomes C. B. F. Woo S. B. Ramsey M. R. Chen X. L. Xu S. (2018). Biological significance of 5-hydroxymethylcytosine in oral epithelial dysplasia and oral squamous cell carcinoma. Oral Surg. Oral Med. Oral Pathol. Oral Radiol. 125 (1), 59–73. 10.1016/j.oooo.2017.06.006 28743666

[B7] Cui X. L. Nie J. Ku J. Dougherty U. West-Szymanski D. C. Collin F. (2020). A human tissue map of 5-hydroxymethylcytosines exhibits tissue specificity through gene and enhancer modulation. Nat. Commun. 11 (1), 6161. 10.1038/s41467-020-20001-w 33268789 PMC7710742

[B8] Ficz G. Branco M. R. Seisenberger S. Santos F. Krueger F. Hore T. A. (2011). Dynamic regulation of 5-hydroxymethylcytosine in mouse ES cells and during differentiation. Nature 473 (7347), 398–402. 10.1038/nature10008 21460836

[B9] Greco C. M. Kunderfranco P. Rubino M. Larcher V. Carullo P. Anselmo A. (2016). DNA hydroxymethylation controls cardiomyocyte gene expression in development and hypertrophy. Nat. Commun. 7, 12418. 10.1038/ncomms12418 27489048 PMC4976219

[B10] Hahn M. A. Qiu R. Wu X. Zhang H. Wang J. Jui J. (2013). Dynamics of 5-hydroxymethylcytosine and chromatin marks in Mammalian neurogenesis. Cell Rep. 3 (2), 291–300. 10.1016/j.celrep.2013.01.011 23403289 PMC3582786

[B11] He Y. F. Li B. Z. Li Z. Liu P. Wang Y. Tang Q. (2011). Tet-mediated formation of 5-carboxylcytosine and its excision by TDG in mammalian DNA. Science 333 (6047), 1303–1307. 10.1126/science.1210944 21817016 PMC3462231

[B12] Ito S. D'Alessio A. C. Taranova O. V. Hong K. Sowers L. C. Zhang Y. (2010). Role of Tet proteins in 5mC to 5hmC conversion, ES-cell self-renewal and inner cell mass specification. Nature 466 (7310), 1129–1133. 10.1038/nature09303 20639862 PMC3491567

[B13] Jäwert F. Hasseus B. Kjeller G. Magnusson B. Sand L. Larsson L. (2013). Loss of 5-hydroxymethylcytosine and TET2 in oral squamous cell carcinoma. Anticancer Res. 33 (10), 4325–4328. 24122999

[B14] Jin B. Li Y. Robertson K. D. (2011a). DNA methylation: superior or subordinate in the epigenetic hierarchy? Genes Cancer 2 (6), 607–617. 10.1177/1947601910393957 21941617 PMC3174260

[B15] Jin S. G. Kadam S. Pfeifer G. P. (2010). Examination of the specificity of DNA methylation profiling techniques towards 5-methylcytosine and 5-hydroxymethylcytosine. Nucleic Acids Res. 38 (11), e125. 10.1093/nar/gkq223 20371518 PMC2887978

[B16] Jin S. G. Wu X. Li A. X. Pfeifer G. P. (2011b). Genomic mapping of 5-hydroxymethylcytosine in the human brain. Nucleic Acids Res. 39 (12), 5015–5024. 10.1093/nar/gkr120 21378125 PMC3130285

[B17] Koh K. P. Yabuuchi A. Rao S. Huang Y. Cunniff K. Nardone J. (2011). Tet1 and Tet2 regulate 5-hydroxymethylcytosine production and cell lineage specification in mouse embryonic stem cells. Cell Stem Cell 8 (2), 200–213. 10.1016/j.stem.2011.01.008 21295276 PMC3134318

[B18] Kohli R. M. Zhang Y. (2013). TET enzymes, TDG and the dynamics of DNA demethylation. Nature 502 (7472), 472–479. 10.1038/nature12750 24153300 PMC4046508

[B19] Kudo Y. Tateishi K. Yamamoto K. Yamamoto S. Asaoka Y. Ijichi H. (2012). Loss of 5-hydroxymethylcytosine is accompanied with malignant cellular transformation. Cancer Sci. 103 (4), 670–676. 10.1111/j.1349-7006.2012.02213.x 22320381 PMC7659252

[B20] Langevin S. M. Butler R. A. Eliot M. Pawlita M. Maccani J. Z. J. McClean M. D. (2014). Novel DNA methylation targets in oral rinse samples predict survival of patients with oral squamous cell carcinoma. Oral Oncol. 50 (11), 1072–1080. 10.1016/j.oraloncology.2014.08.015 25242135 PMC4254027

[B21] Larsen F. Gundersen G. Lopez R. Prydz H. (1992). CpG islands as gene markers in the human genome. Genomics 13 (4), 1095–1107. 10.1016/0888-7543(92)90024-m 1505946

[B22] Lian C. G. Xu Y. Ceol C. Wu F. Larson A. Dresser K. (2012). Loss of 5-hydroxymethylcytosine is an epigenetic hallmark of melanoma. Cell 150 (6), 1135–1146. 10.1016/j.cell.2012.07.033 22980977 PMC3770275

[B23] Lister R. Mukamel E. A. Nery J. R. Urich M. Puddifoot C. A. Johnson N. D. (2013). Global epigenomic reconfiguration during mammalian brain development. Science 341 (6146), 1237905. 10.1126/science.1237905 23828890 PMC3785061

[B24] Liu S. de Medeiros M. C. Fernandez E. M. Zarins K. R. Cavalcante R. G. Qin T. (2020). 5-Hydroxymethylation highlights the heterogeneity in keratinization and cell junctions in head and neck cancers. Clin. Epigenetics 12 (1), 175. 10.1186/s13148-020-00965-8 33203436 PMC7672859

[B25] Luo C. Hajkova P. Ecker J. R. (2018). Dynamic DNA methylation: in the right place at the right time. Science 361 (6409), 1336–1340. 10.1126/science.aat6806 30262495 PMC6197482

[B26] Lyko F. (2018). The DNA methyltransferase family: a versatile toolkit for epigenetic regulation. Nat. Rev. Genet. 19 (2), 81–92. 10.1038/nrg.2017.80 29033456

[B27] Mariani C. J. Vasanthakumar A. Madzo J. Yesilkanal A. Bhagat T. Yu Y. (2014). TET1-mediated hydroxymethylation facilitates hypoxic gene induction in neuroblastoma. Cell Rep. 7 (5), 1343–1352. 10.1016/j.celrep.2014.04.040 24835990 PMC4516227

[B28] Pastor W. A. Pape U. J. Huang Y. Henderson H. R. Lister R. Ko M. (2011). Genome-wide mapping of 5-hydroxymethylcytosine in embryonic stem cells. Nature 473 (7347), 394–397. 10.1038/nature10102 21552279 PMC3124347

[B29] Pickering C. R. Zhang J. Yoo S. Y. Bengtsson L. Moorthy S. Neskey D. M. (2013). Integrative genomic characterization of oral squamous cell carcinoma identifies frequent somatic drivers. Cancer Discov. 3 (7), 770–781. 10.1158/2159-8290.CD-12-0537 23619168 PMC3858325

[B30] Piyathilake C. J. Bell W. C. Jones J. Henao O. L. Heimburger D. C. Niveleau A. (2005). Pattern of nonspecific (or global) DNA methylation in oral carcinogenesis. Head. Neck 27 (12), 1061–1067. 10.1002/hed.20288 16155917 PMC1853326

[B31] Qu J. Zhou M. Song Q. Hong E. E. Smith A. D. (2013). MLML: consistent simultaneous estimates of DNA methylation and hydroxymethylation. Bioinformatics 29 (20), 2645–2646. 10.1093/bioinformatics/btt459 23969133 PMC3789553

[B32] Rechache N. S. Wang Y. Stevenson H. S. Killian J. K. Edelman D. C. Merino M. (2012). DNA methylation profiling identifies global methylation differences and markers of adrenocortical tumors. J. Clin. Endocrinol. Metab. 97 (6), E1004–E1013. 10.1210/jc.2011-3298 22472567 PMC3387424

[B33] Smith Z. D. Meissner A. (2013). DNA methylation: roles in mammalian development. Nat. Rev. Genet. 14 (3), 204–220. 10.1038/nrg3354 23400093

[B34] Song C. X. Szulwach K. E. Dai Q. Fu Y. Mao S. Q. Lin L. (2013). Genome-wide profiling of 5-formylcytosine reveals its roles in epigenetic priming. Cell 153 (3), 678–691. 10.1016/j.cell.2013.04.001 23602153 PMC3657391

[B35] Song C. X. Szulwach K. E. Fu Y. Dai Q. Yi C. Li X. (2011). Selective chemical labeling reveals the genome-wide distribution of 5-hydroxymethylcytosine. Nat. Biotechnol. 29 (1), 68–72. 10.1038/nbt.1732 21151123 PMC3107705

[B36] Spruijt C. G. Gnerlich F. Smits A. H. Pfaffeneder T. Jansen P. W. T. C. Bauer C. (2013). Dynamic readers for 5-(hydroxy)methylcytosine and its oxidized derivatives. Cell 152 (5), 1146–1159. 10.1016/j.cell.2013.02.004 23434322

[B37] Stroud H. Feng S. Morey Kinney S. Pradhan S. Jacobsen S. E. (2011). 5-Hydroxymethylcytosine is associated with enhancers and gene bodies in human embryonic stem cells. Genome Biol. 12 (6), R54. 10.1186/gb-2011-12-6-r54 21689397 PMC3218842

[B38] Takayama K. Misawa A. Suzuki T. Takagi K. Hayashizaki Y. Fujimura T. (2015). TET2 repression by androgen hormone regulates global hydroxymethylation status and prostate cancer progression. Nat. Commun. 6, 8219. 10.1038/ncomms9219 26404510

[B39] Tang Z. Kang B. Li C. Chen T. Zhang Z. (2019). GEPIA2: an enhanced web server for large-scale expression profiling and interactive analysis. Nucleic Acids Res. 47 (W1), W556–W560. 10.1093/nar/gkz430 31114875 PMC6602440

[B40] Towle R. Truong D. Hogg K. Robinson W. P. Poh C. F. Garnis C. (2013). Global analysis of DNA methylation changes during progression of oral cancer. Oral Oncol. 49 (11), 1033–1042. 10.1016/j.oraloncology.2013.08.005 24035722

[B41] Wan Z. Xiong H. Tan X. Su T. Xia K. Wang D. (2021). Integrative multi-omics analysis reveals candidate biomarkers for oral squamous cell carcinoma. Front. Oncol. 11, 794146. 10.3389/fonc.2021.794146 35096593 PMC8795899

[B42] Wang Y. Hu H. Wang Q. Li Z. Zhu Y. Zhang W. (2017). The level and clinical significance of 5-hydroxymethylcytosine in oral squamous cell carcinoma: an immunohistochemical study in 95 patients. Pathol. Res. Pract. 213 (8), 969–974. 10.1016/j.prp.2017.04.016 28554766

[B43] Wernig-Zorc S. Yadav M. P. Kopparapu P. K. Bemark M. Kristjansdottir H. L. Andersson P. O. (2019). Global distribution of DNA hydroxymethylation and DNA methylation in chronic lymphocytic leukemia. Epigenetics Chromatin 12 (1), 4. 10.1186/s13072-018-0252-7 30616658 PMC6322269

[B44] Wu H. D'Alessio A. C. Ito S. Wang Z. Cui K. Zhao K. (2011). Genome-wide analysis of 5-hydroxymethylcytosine distribution reveals its dual function in transcriptional regulation in mouse embryonic stem cells. Genes Dev. 25 (7), 679–684. 10.1101/gad.2036011 21460036 PMC3070931

[B45] Wu H. Zhang Y. (2014). Reversing DNA methylation: mechanisms, genomics, and biological functions. Cell 156 (1-2), 45–68. 10.1016/j.cell.2013.12.019 24439369 PMC3938284

[B46] Wu X. Zhang Y. (2017). TET-Mediated active DNA demethylation: mechanism, function and beyond. Nat. Rev. Genet. 18 (9), 517–534. 10.1038/nrg.2017.33 28555658

[B47] Yang H. Liu Y. Bai F. Zhang J. Y. Ma S. H. Liu J. (2013). Tumor development is associated with decrease of TET gene expression and 5-methylcytosine hydroxylation. Oncogene 32 (5), 663–669. 10.1038/onc.2012.67 22391558 PMC3897214

[B48] Yildirim O. Li R. Hung J. H. Chen P. B. Dong X. Ee L. S. (2011). Mbd3/NURD complex regulates expression of 5-hydroxymethylcytosine marked genes in embryonic stem cells. Cell 147 (7), 1498–1510. 10.1016/j.cell.2011.11.054 22196727 PMC3252821

[B49] Yu M. Hon G. C. Szulwach K. E. Song C. X. Zhang L. Kim A. (2012). Base-resolution analysis of 5-hydroxymethylcytosine in the mammalian genome. Cell 149 (6), 1368–1380. 10.1016/j.cell.2012.04.027 22608086 PMC3589129

