# Supplementary figures and images for: Unidirectional alteration of methylation and hydroxymethylation at the promoters and differential gene expression in oral squamous cell carcinoma
Source: Front Genet. 2023 Oct 12;14:1269084. doi: 10.3389/fgene.2023.1269084 (PMC10603190; doi:10.3389/fgene.2023.1269084)

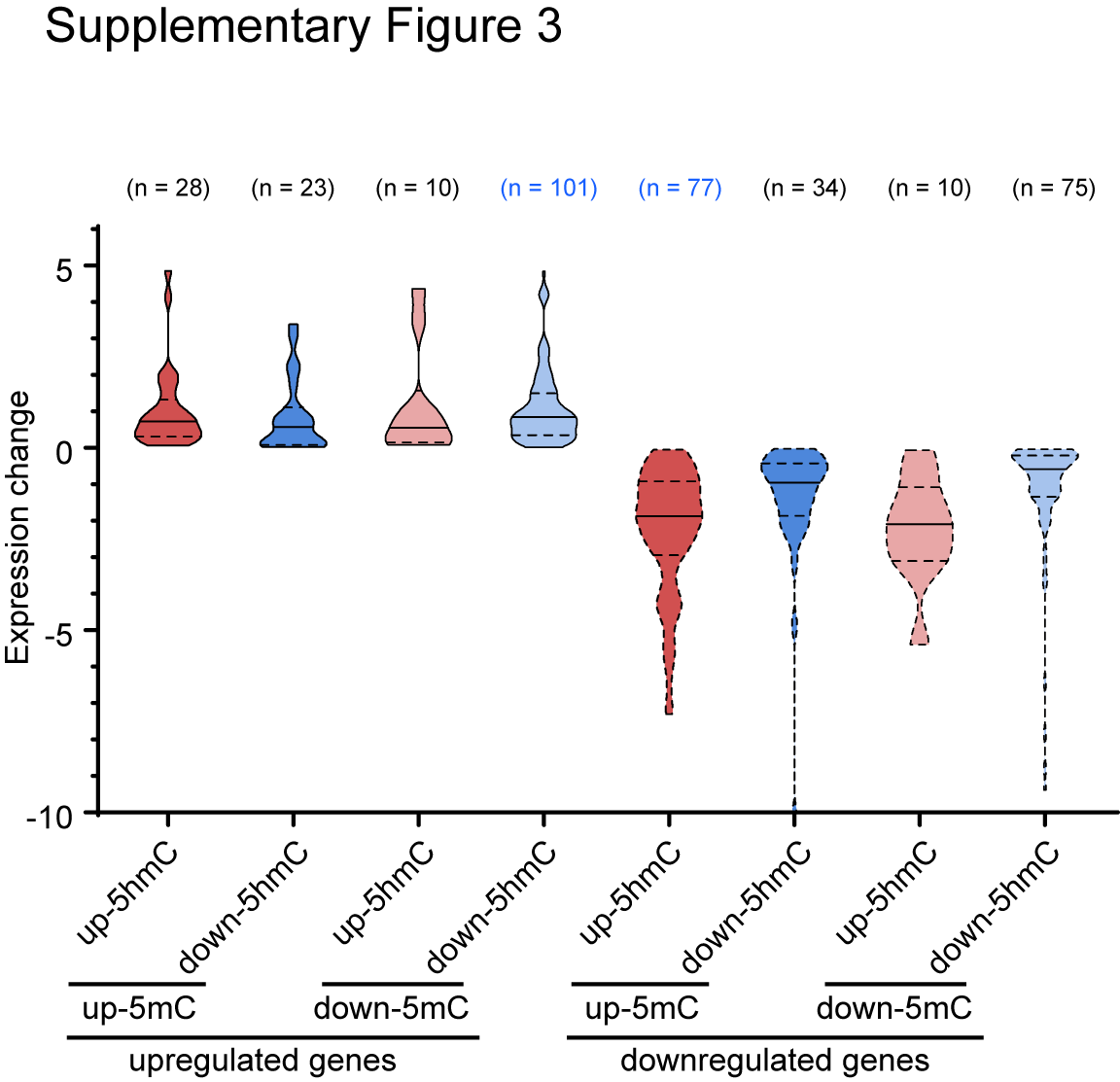

Supplement: Supplementary file 3 [file Image3.TIF]

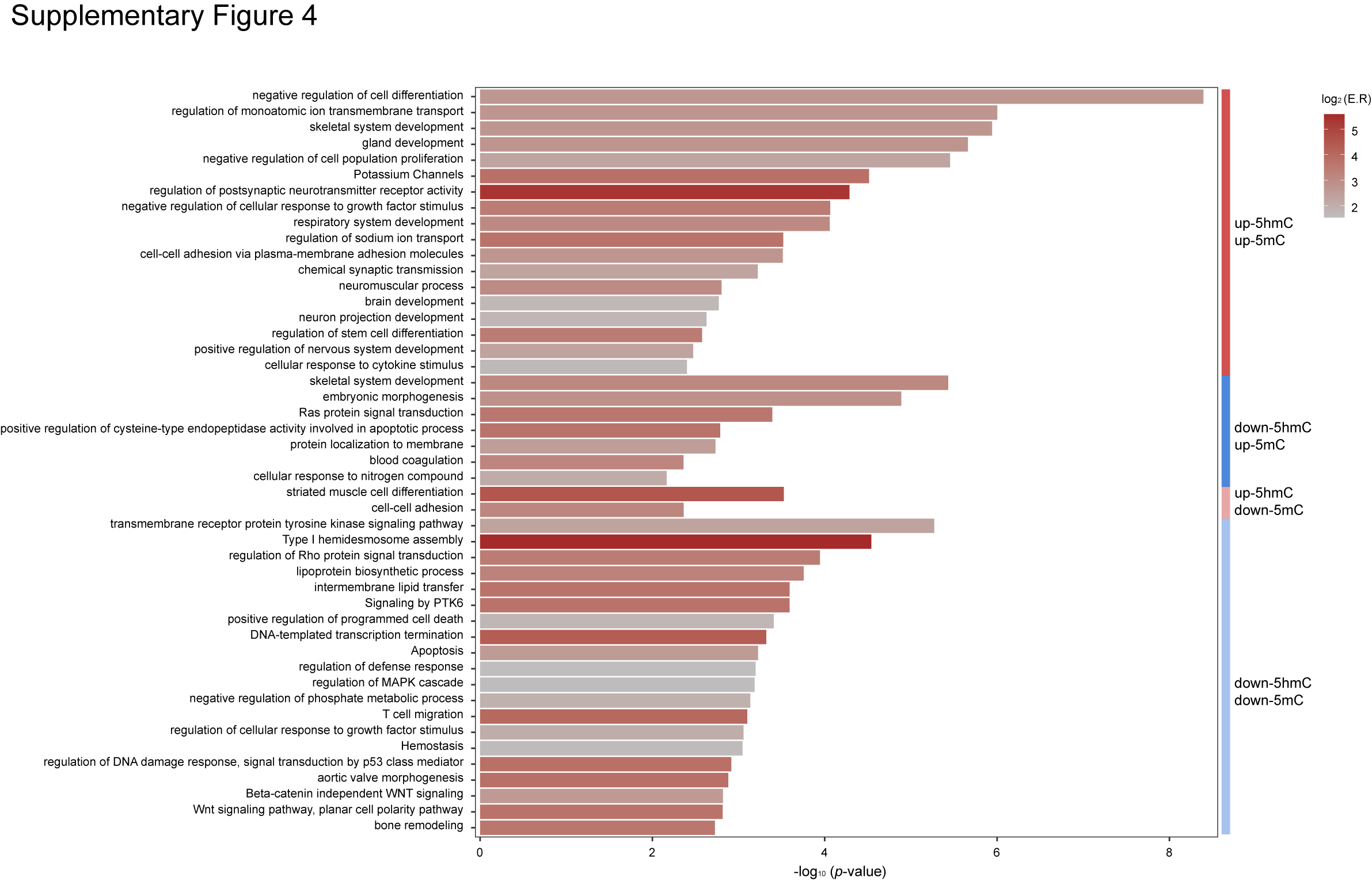

Supplement: Supplementary file 4 [file Image4.TIF]

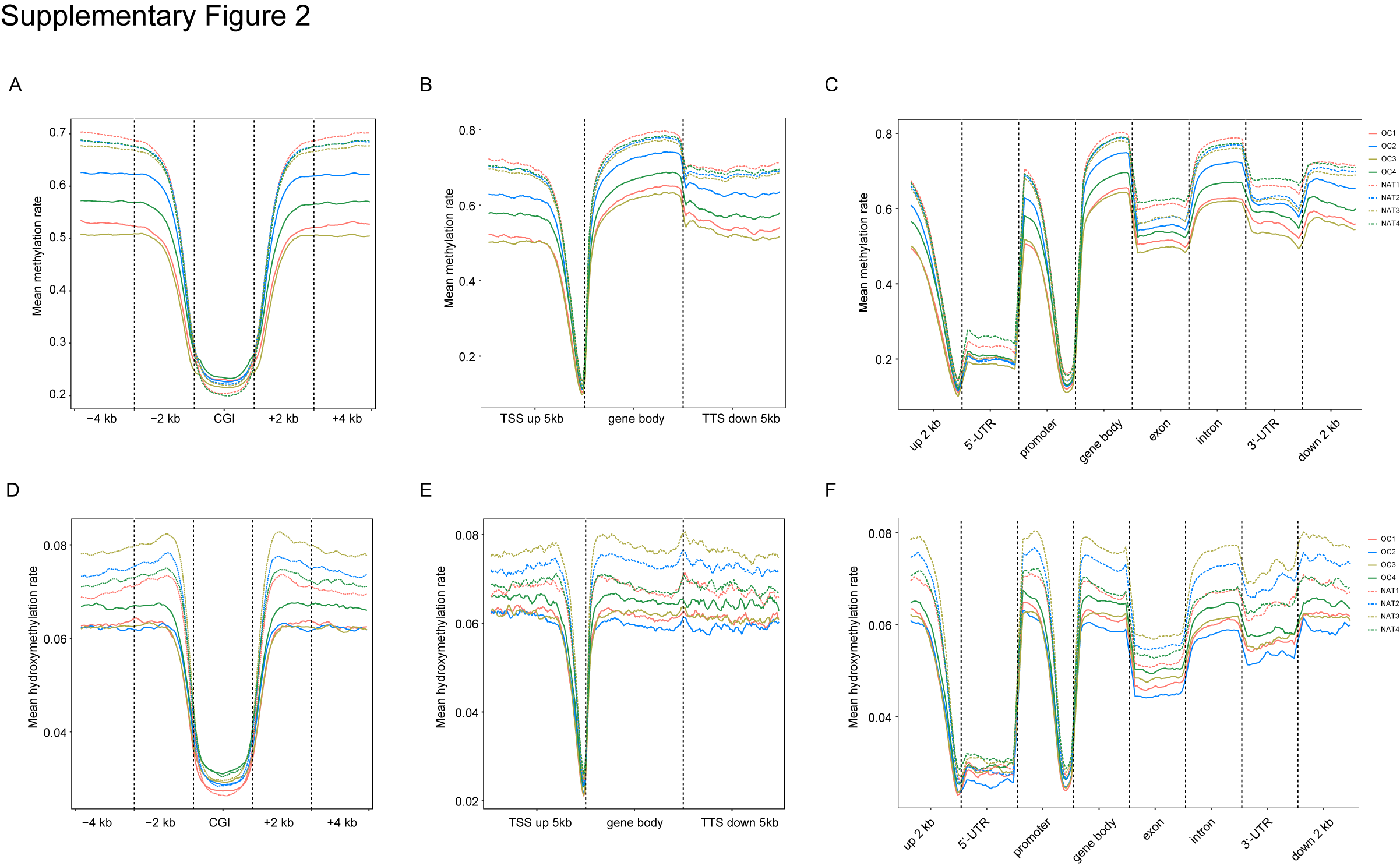

Supplement: Supplementary file 5 [file Image2.TIF]

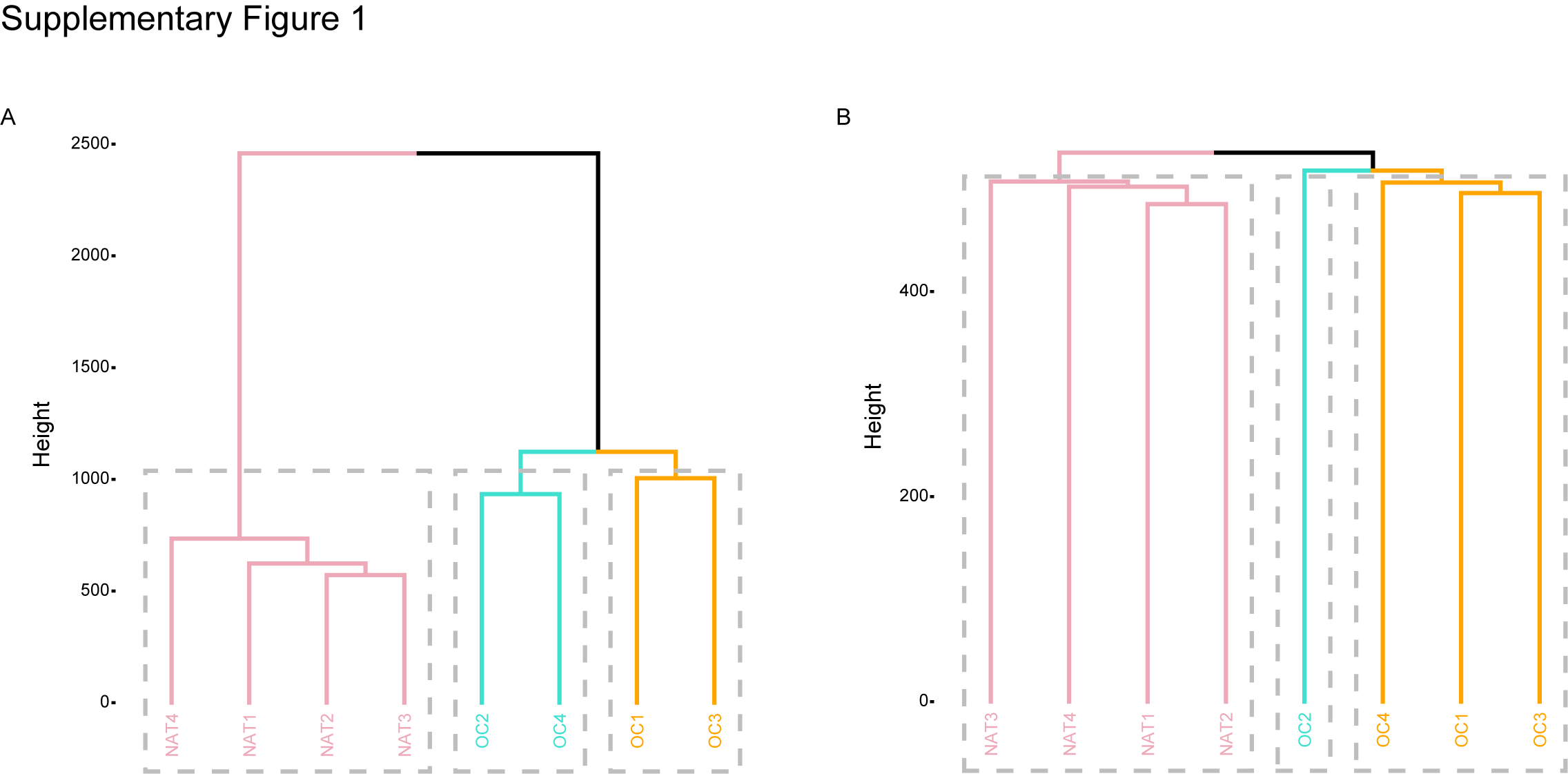

Supplement: Supplementary file 6 [file Image1.TIF]
